# Intraocular pressure change during reading or writing on smartphone

**DOI:** 10.1371/journal.pone.0206061

**Published:** 2018-10-25

**Authors:** Ahnul Ha, Young Kook Kim, Young Joo Park, Jin Wook Jeoung, Ki Ho Park

**Affiliations:** 1 Department of Ophthalmology, Seoul National University College of Medicine, Seoul, Korea; 2 Department of Ophthalmology, Seoul National University Hospital, Seoul, Korea; 3 Department of Ophthalmology, Seoul National University Bundang Hospital, Seongnam, Korea; Bascom Palmer Eye Institute, UNITED STATES

## Abstract

**Purpose:**

To investigate the effect of reading or writing on a smartphone in terms of intraocular pressure (IOP) changes.

**Design:**

Prospective, comparative case series.

**Participants:**

Thirty-nine (39) healthy young (age < 40) volunteers.

**Methods:**

The participants were requested to conduct standardized work (i.e., read a sample text on single mobile device and subsequently type it on the same device) under *daylight* [300 lux] and *low-light* [100 lux] conditions independently on consecutive days. On each day, three sets of IOP measurements (total: 7) using a rebound tonometer (iCare PRO; Tiolat, Helsinki, Finland) were performed: (1) *pre-work* (baseline) [2 measurements], (2) *during smartphone work* [5, 15, and 25 minutes], and (3) *post-work* [5 and 15 minutes].

**Main outcome measures:**

Changes in IOP at different check-out points.

**Results:**

Under the daylight condition, the mean baseline IOP was 13.7 ± 1.8 mmHg, and the mean IOP increased after 5 minutes of work (14.1 ± 1.8 mmHg; +2.0 ± 1.9%; *P* < 0.001). When the smartphone work lasted for 15 minutes, the IOP showed a further significant increase (15.5 ± 1.7 mmHg; +12.9 ± 4.4%; *P* < 0.001), which persisted over the course of the 25 minutes of smartphone work (15.3 ± 1.8 mmHg; +11.1 ± 3.9%; *P* < 0.001); then, after stopping work for 5 minutes, the IOP was restored (13.9 ± 1.7 mmHg; +0.9 ± 2.1%; *P* = 0.220). Under the low-light condition, the mean IOP was significantly increased immediately after 5 minutes of smartphone work (from 13.9 ± 1.9 to 15.6 ± 1.8 mmHg; +12.1 ± 4.8%; *P* < 0.001); this IOP increase continued: 17.3 ± 1.9 [+24.7 ± 10.3%] at 15 minutes’ work, and 17.0 ± 1.7 mmHg [+23.1 ± 9.5%] at 25 minutes’ work (*P* < 0.001 at both check-out points). Five minutes after stopping the smartphone work, interestingly, the IOP significantly dropped, to a level even lower than that of the pre-work (12.8 ± 1.9 mmHg; -8.1 ± 3.0%; *P* < 0.001), and at post-work 15 minutes, the IOP returned to the baseline (13.9 ± 1.8 mmHg; -0.3 ± 2.6%; *P* = 0.360).

**Conclusions:**

In healthy young subjects, reading or writing on smartphone significantly increased IOP, and the changes of IOP were faster and greater under the low-light condition. Smartphone users who are concerned about IOP fluctuation are advised to (1) take a break if they read or write on smartphone for more than 5 minutes, and (2) avoid using smartphones wherever possible in dark places.

## Introduction

Personal mobile devices (i.e., smartphones) have become a huge part of daily living. The latest devices are so advanced that they are more like hand-held computers. Accordingly, many people spend a considerable amount of time using smartphones: not only for phone calls but also for daily activities such as checking email and watching movies. Significantly, it is likely that smartphone dependences such as these will increase.

Given the substantial number of hours being devoted to the viewing of electronic devices, it is concerning that the frequency of occurrence and magnitude of ophthalmologic symptoms is significantly higher when using digital displays compared with hard-copy printed materials [1]. Ocular complaints such as eye strain, irritation and blurred vision experienced by electronic-device users are now known, universally as Computer Vision Syndrome (CVS) [2].

Sustained effort for accommodation and the accompanying ocular-surface-related changes such as dry eye are among the widely proposed mechanisms for CVS. Ocular changes related to near vision are expected to cause temporary but meaningful fluctuations in intraocular pressure (IOP) as well. Yan et al. reported that accommodation could induce transient IOP elevation in those with progressing myopia [3]. Qudsiya et al. showed that 4 hours of computer work results in significant IOP elevation among healthy young individuals [4]. However, the exact mechanism and associated structural modifications causing IOP increase have not been thoroughly studied.

The unique aspects of smartphones in relation to conventional computers, such as screen size, resolution, contrast, image refresh rates, and screen glare, are expected to have distinctive effects on the eyes. Certainly too, the viewing distance and angle of smartphones are not the same as those of computer monitors; and due to the portability of smartphones, a considerable number of people use them under dim illumination. In terms of IOP change accompanying smartphone work (i.e., reading or writing), furthermore, more various factors are expected to be related.

Thus prompted, we investigated the effect of reading or writing on a smartphone in terms of the pattern and magnitude of IOP change. This prospective, comparative case series study was conducted with healthy young volunteers in daylight and low-light condition independently on consecutive days.

## Methods

This study was approved by the Seoul National University Hospital Institutional Review Board and adhered to the tenets of the Declaration of Helsinki. All of the participants provided written informed consent.

### Study subjects

This study was conducted on healthy volunteers who had visited Seoul National University Hospital’s Glaucoma Clinic from March 2017 to June 2017 for routine ophthalmologic examinations. Prior to commencement of the experimentation, all subjects underwent a complete ophthalmic examination: visual acuity assessment, refraction, slit-lamp biomicroscopy, gonioscopy, Goldmann applanation tonometry (Haag-Streit, Koniz, Switzerland), and dilated-funduscopic examination. Additionally, they underwent the following: central corneal thickness measurement (CCT: Orbscan 73 II, Bausch & Lomb Surgical, Rochester, NY, USA), digital color stereo disc photography, red-free retinal nerve fiber layer photography.

Upon enrollment in the study, the eligible participants were under the age of 40, had normal near vision in both eyes and showed no abnormal findings in any previous ophthalmic examinations. Patients were excluded from further analysis for any of the following reasons: history of any ocular pathology; intraocular surgery or significant trauma; any degree of tropia/phoria greater than 5 prism diopters; any associated systemic disease. When both eyes of a subject were eligible, only right eyes were selected for inclusion in the study.

### Sample size estimation

Sample size calculation was performed using the Power and Sample Size Program software (version 3.1.2, available at: http://biostat.mc.vanderbilt.edu/wiki/Main/PowerSampleSize). According to the previous research, the average baseline IOP of the study group was expected to be around 13.0 mmHg [5]. Based on our pilot study, a mean IOP divergence from the baseline after smartphone usage was assumed to be 1.8 mmHg. With an alpha error of 0.05 and 90% of power, the minimum adequate sample size was 22 for each light condition, according to an expected maximum standard deviation of IOP difference in the matched pairs of 2.5 mmHg.

### Protocols of smartphone work

A single smartphone (Samsung Galaxy S6, Samsung Electronics Co., Ltd., Seoul, Korea, 5.1 inch screen, 577 ppi pixel resolution) with a customized brightness setting (432 cd/m^2^) was used. The participants were requested to perform a standardized work (i.e., read a sample text on a smartphone and subsequently type it on the same device) with their habitual posture. At the beginning of the smartphone work, the viewing distance (from the smartphone to the subject’s right eye) and head posture (difference of angle between upright head position and viewing head posture) were evaluated. During the smartphone usage, participants were asked to continuously maintain the same distance and head posture.

On the smartphone screen, an article in English was presented. The text font was 1.5 mm in size (i.e., the vertical height of a lowercase letter without ascenders or descenders, as measured through a +20 D lens with a ruler [6]) and of black color on a white background. The lines were double-spaced. In order to maintain concentration on the smartphone screen, the participants were asked to continuously type the presented sentences on the smartphone screen. The brightness of the smartphone display was, as indicated above, fixed throughout the study.

**Fig 1:**
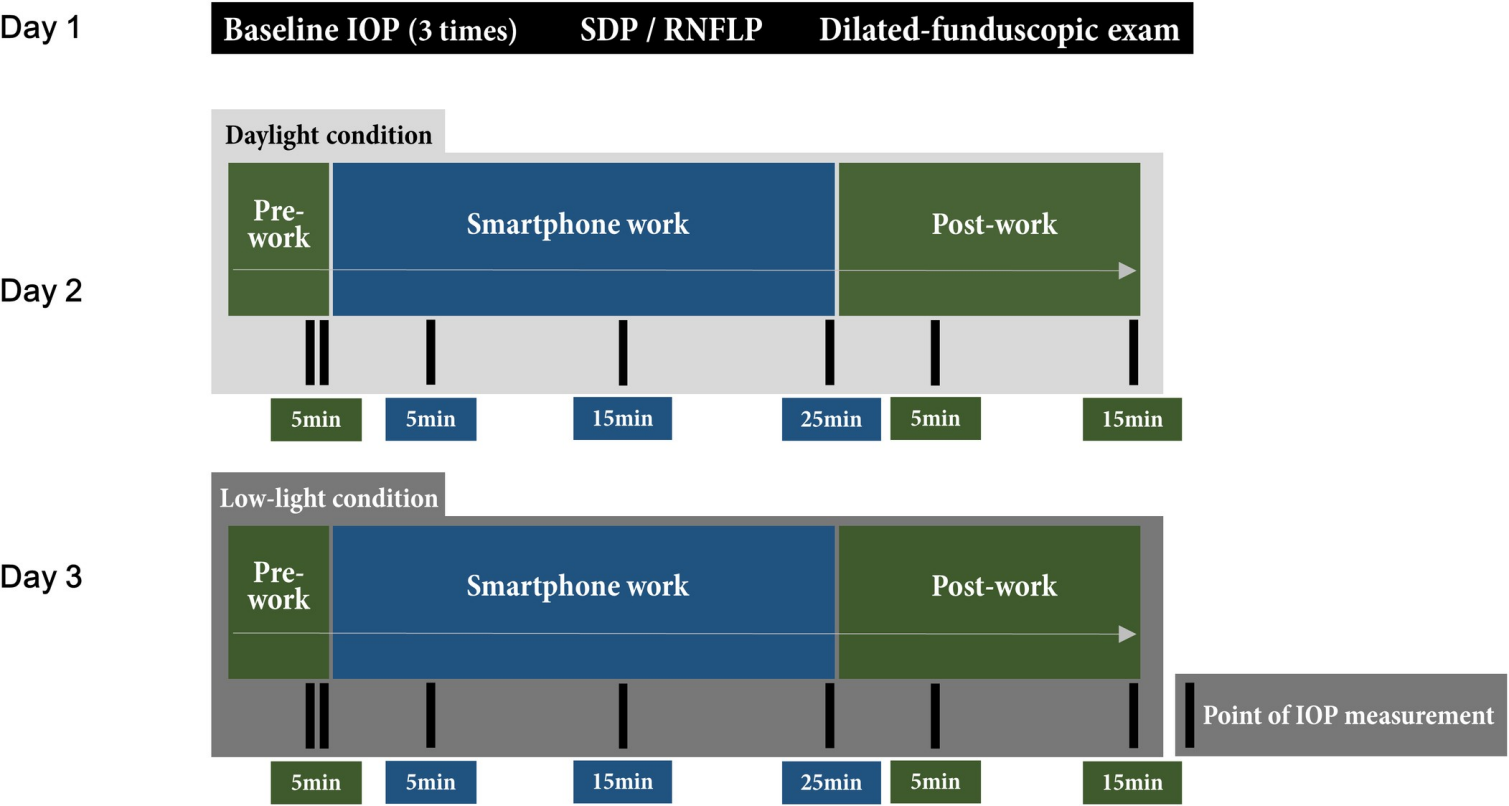
Schematic representation of study protocol. The baseline ophthalmologic examination and smartphone work under daylight and low-light conditions were performed independently on consecutive days.

The experiments were conducted in daylight (luminance level: 300 lux) and low-light (luminance level: 100 lux) condition independently on consecutive days. A flowchart of the study plan is provided in Fig 1. The daylight and low-light experiments were performed as close to the same hour of the day as possible.

### Measurements of IOP

All of the IOPs were measured by an experienced glaucoma specialist (AH) using a hand-held, anesthetic-free rebound tonometer (iCare PRO; Tiolat, Helsinki, Finland). To avoid investigator bias, an independent observer (YJP) read the pressures without the investigator’s knowledge. During the IOP measurements, all of the participants maintained an upright sitting posture and a contra-lateral-eye view of the smartphone screen. The IOP reading was obtained by the contact of the tip of the probe with the central cornea, without any eyelid manipulation. Each IOP value was measured in the series mode: the final IOP was calculated by taking 6 automatic measurements, discarding the highest and lowest readings, and averaging the remaining 4 readings [7].

Under both the daylight and low-light conditions, the subjects underwent 3 sets of IOP measurements: (1) pre-work (baseline) [2 measurements], (2) during smartphone work [5, 15 and 25 minutes], and (3) post-work [5 and 15 minutes]. The pre-work IOP was measured after 5 minutes of daylight or low-light adaptation, and the average value of the two measurement series was recorded as the final pre-work IOP. The change of IOP during and after smartphone usage was expressed as the percentage change from the baseline (pre-work) value.

### Statistical analysis

Longitudinal changes in IOP were examined by comparison between pre-work and each interval value using the repeated-measures analysis of variance (ANOVA). Then, the extents of IOP change at different time points were compared using the paired t-test with Bonferroni correction. Pearson correlation analysis was performed to evaluate the relationship between the percentage change of IOP and the other relevant factors (age, spherical equivalent, smartphone viewing distance, and angle of neck flexion). The statistical analysis was performed using the SPSS statistical package (SPSS 22.0; Chicago, IL, USA). A 2-sided *P* value less than 0.05 was considered to be statistically significant, unless the Bonferroni correction method for multiple comparisons was used, in which case a value less than 0.01 was the standard.

## Results

A total of 39 participants (39 eyes) were recruited for the current study. Their mean age was 28.6 ± 4.4 (range: 22–38) years; 21 were men (53.8%) and 18 women (46.2%). Participants’ demographic and clinical data are summarized in Table 1.

**Table 1:** Clinical characteristics of participants.

	Participants (*n* = 39)
Age (yrs)	28.6 ± 4.4
Gender (F / M)	18 / 21
Spherical equivalent (D)	-1.1 ± 1.7
Baseline IOP* (mmHg)	13.5 ± 1.9
CCT (mm)	0.542 ± 0.021

Values are mean ± standard deviation.

*Baseline IOP: IOP measured by Goldmann applanation tonometry.

F, female; M, male; D, diopters; CCT, central corneal thickness.

The pre-work mean IOP levels were 13.7 ± 1.8 (range: 10.0–17.1) and 13.9 ± 1.9 (range: 10.1–17.5) mmHg at daylight and low-light condition, respectively. No significant differences under the two light conditions in terms of pre-work IOP were found (*P* = 0.062).

**Fig 2:**
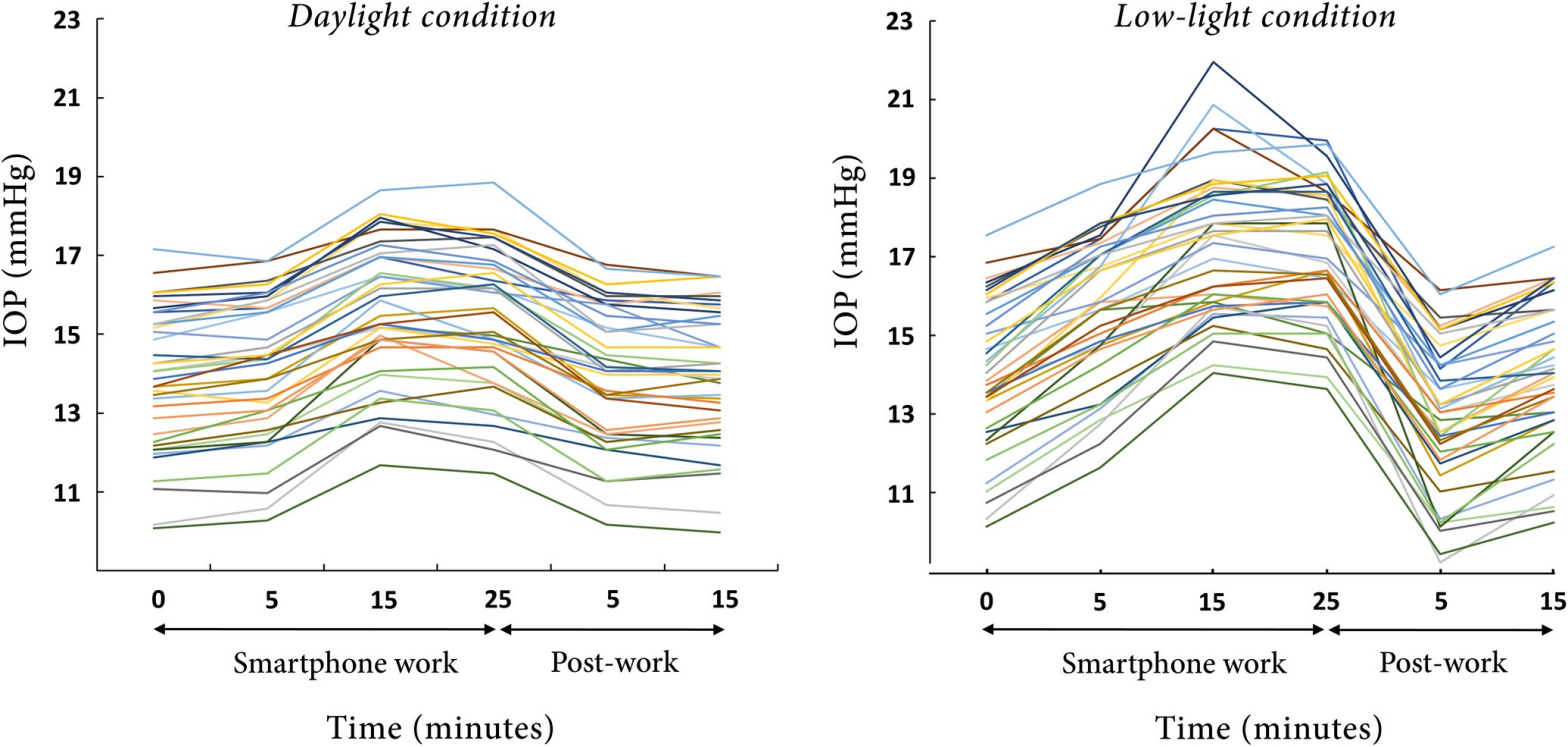
**Graph depicting changes of intraocular pressure (IOP) among group of 39 eyes during and after smartphone work under daylight (A) and low-light (B) conditions.** Note that the X-axis is not to scale but depicts the times at which the IOPs were measured during and after the smartphone work.

**Fig 3:**
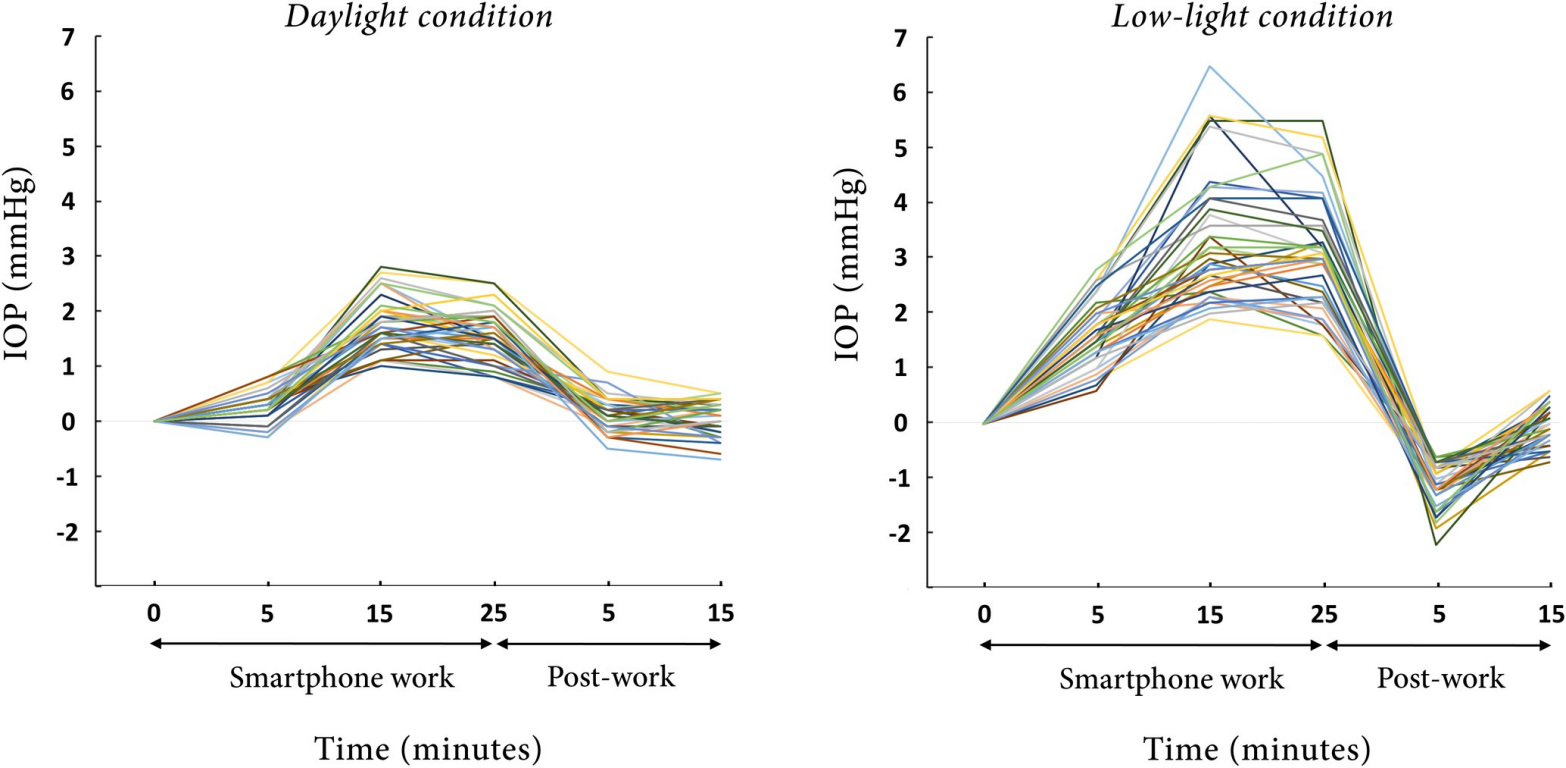
Graph showing intraocular pressure (IOP) fluctuation during and after smartphone work, with pre-work IOP values in all participants converted to zero (0). (A) daylight condition and (B) low-light condition. Note that the X-axis is not to scale but depicts the times at which the IOPs were measured during and after the smartphone work.

**Fig 4:**
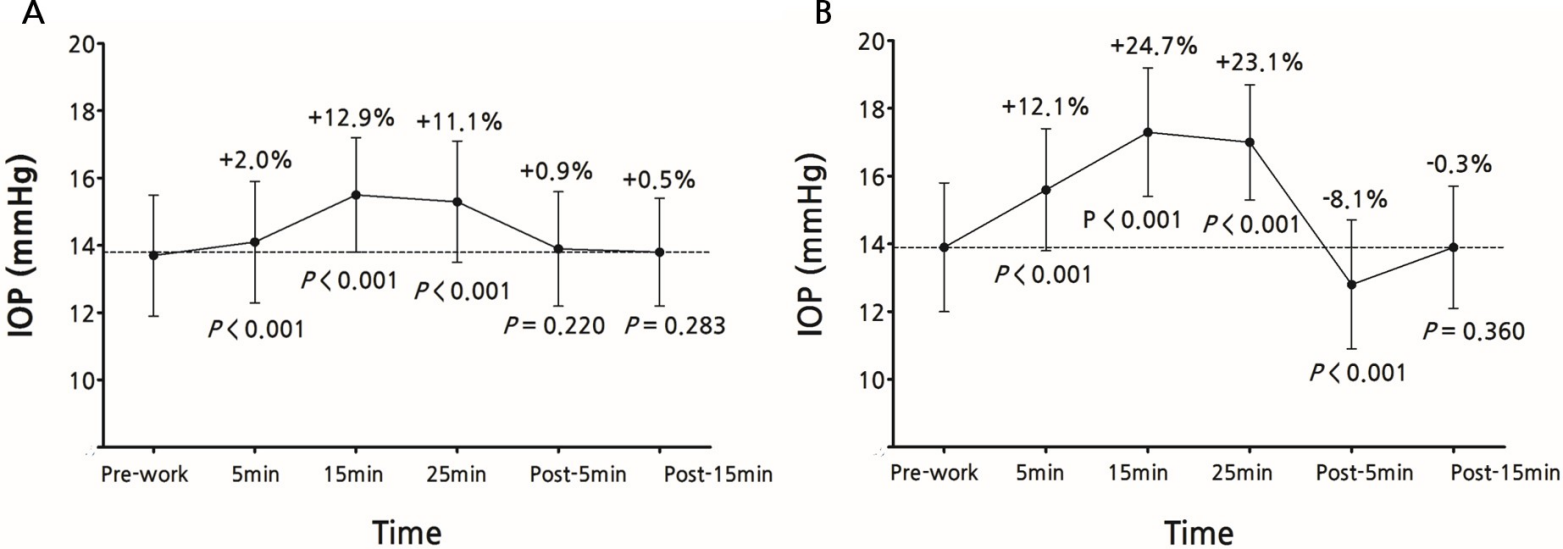
**Mean intraocular pressure (IOP) of 39 eyes during and after smartphone work under daylight (A) and low-light (B) conditions.** Daylight condition: mean IOPs significantly rose after 5 minutes of smartphone work (*P* < 0.001), and were sustained until 25 minutes of smartphone use (*P* < 0.001); within 5 minutes after stopping the smartphone work, the IOP returned to the pre-work level. Low-light condition: mean IOPs were elevated during 5, 15 and 25 minutes of smartphone work (*P* < 0.001 at all points); 5 minutes after stopping the smartphone work, the IOP dropped below the pre-work level (*P* < 0.001); 15 minutes after the work cessation, the IOP returned to the pre-work level. Note that the X-axis is not to scale but depicts the times at which the IOPs were measured during and after the smartphone work. The Y error bars indicate the standard error of the mean.

Under the daylight condition, the mean IOP increased after 5 minutes of work (14.1 ± 1.8 mmHg; +2.0 ± 1.9%; *P* < 0.001). After working on the smartphone for 15 minutes, the mean IOP increased further (15.5 ± 1.7 mmHg; +12.9 ± 4.4%; *P* < 0.001), which increase persisted over the course of the 25 minutes of smartphone work (15.3 ± 1.8 mmHg; +11.1 ± 3.9%; *P* < 0.001). After stopping the smartphone work, the IOP returned to the pre-work level. The mean IOPs were 13.9 ± 1.7 and 13.8 ± 1.6 mmHg for post-work 5 and 15 minutes, respectively (+0.9 ± 2.1%; *P* = 0.220 and +0.5 ± 2.2%; *P* = 0.283; Figs 2A, 3A and 4A).

Under the low-light condition, the mean IOP significantly increased immediately after 5 minutes of smartphone work (15.6 ± 1.8 mmHg; *P* < 0.001); this change represented a 12.1 ± 4.8% increment from the pre-work value. The elevated IOP continued throughout the work: at 15 and 25 minutes, the mean IOP readings were 17.3 ± 1.9 (+24.7 ± 10.3%) and 17.0 ± 1.7 (+23.1 ± 9.5%) mmHg, respectively. The degrees of IOP increment were statistically significant (*P* < 0.001 at both check-out points). Interestingly, 5 minutes after stopping the work, the mean IOP dropped below the pre-work level (12.8 ± 1.9 mmHg; *P* < 0.001); this difference in IOP represented an 8.1 ± 3.0% IOP reduction. The IOP returned to the pre-work level 15 minutes after the cessation of the smartphone work (13.9 ± 1.8 mmHg; -0.3 ± 2.6%; *P* = 0.360; Figs 2B, 3B and 4B). Table 2 summarizes the IOP changes during the smartphone work.

**Table 2:** IOP changes of study participants.

		Pre-work	5 min	15 min	25 min	Post-5 min	Post-15 min
Daylight	IOP (mmHg)	13.7 ± 1.8	14.1 ± 1.8	15.5 ± 1.7	15.3 ± 1.8	13.9 ± 1.7	13.8 ± 1.6
	ΔIOP (%)		+2.0 ± 1.9	+12.9 ± 4.4	+11.1 ± 3.9	+0.9 ± 2.1	+0.5 ± 2.2
Low-light	IOP (mmHg)	13.9 ± 1.9	15.6 ± 1.8	17.3 ± 1.9	17.0 ± 1.7	12.8 ± 1.9	13.9 ± 1.8
	ΔIOP (%)		+12.1 ± 4.8	+24.7 ± 10.3	+23.1 ± 9.5	-8.1 ± 3.0	-0.3 ± 2.6

Values are mean ± standard deviation.

ΔIOP (%) was calculated as the difference between the maximum IOP during smartphone work (at 15 or 25 minutes of smartphone work) and the pre-work IOP [= (Maximum IOP–Pre-work IOP) / Pre-work IOP X 100].

Table 3 shows the results of a Pearson correlation analysis performed to evaluate the association between the maximum mean percentage change of IOP (ΔIOP [%]) and the other relevant clinical factors. The maximum mean change of IOP was defined as the difference between the maximum IOP during smartphone work (either at 15 or 25 minutes of work) and the pre-work IOP. Under both daylight and low-light condition, IOP difference showed strong correlation (*r* = 0.379 and *r* = 0.707, respectively) with greater neck-flexion angle (*P* = 0.017 and *P* < 0.001, respectively). The results also shown that the lower the pre-work IOP, the greater the ΔIOP under both daylight and low-light conditions. However, these results are likely to be affected by the fact that pre-work IOP corresponds to a denominator in the formula for obtaining the ΔIOP (%).

**Table 3:** Pearson correlation analysis between clinical parameters and intraocular pressure changes during smartphone task.

Clinical parameter	ΔIOP (%)		
	Illumination	Correlation coefficient	*P*
Age	Low-light	0.019	0.909
	Daylight	0.007	0.968
SE	Low-light	-0.175	0.287
	Daylight	0.074	0.656
Pre-task IOP	Low-light	-0.634	**< 0.001**
	Daylight	-0.596	**< 0.001**
Distance	Low-light	0.286	0.078
	Daylight	0.101	0.539
Head position	Low-light	0.707	**< 0.001**
	Daylight	0.379	**0.017**

IOP, Intraocular pressure

ΔIOP, Maximum percent change of IOP from pre-task value; SE, Spherical equivalent.

Statistically significant *P* values are presented with bold characters.

## Discussion

The present study demonstrated that working on a smartphone can transiently increase IOP under both daylight and low-light conditions; further, it determined that the IOP increased faster, and the extent of IOP fluctuation was greater, under the low-light condition than in the daylight condition.

The underlying ocular dynamics for these IOP changes during and after smartphone work are unclear, though the following mechanisms are assumed to be involved: 1) Accommodation and convergence; 2) external ocular muscle (EOM) contraction; 3) psychophysiological stress; 4) dry eye; 5) neck-flexion posture.

### Accommodation and convergence

Three physiological responses; accommodation, pupil constriction, and convergence occur when the eye tries to focus at near. The change in IOP associated with near work had been investigated by a number of studies. Coleman et al. revealed that near focus induces a small, 2–4 mmHg increase in IOP with a subsequent small decrease of pressure level [8]. Yan et al. reported that the IOPs of young progressing myopic subjects rose with accommodation [3]. Smartphones’ small screen size necessitates reduced font sizes, leading to closer viewing distances, which increases the demands on both accommodation and convergence [6]. Additionally, as the object viewed on a smartphone changes frequently, the eyes need to keep changing focus to maintain a clear image. Given especially the substantial time spent in smartphone viewing, sustained active accommodation and convergence potentially can lead to elevated IOP.

### EOM contraction

When the EOM contracts, the IOP level can be increased [9]. In a previous study utilizing direct-recorded IOP readings, 5–10 mmHg of sustained IOP elevation was produced during levoversion [8]. During smartphone use, specifically while text scrolling and reading, extensive ocular motions with EOM contraction are required. Moreover, as a hand-held device, a smartphone can be positioned in almost any direction, thereby requiring, at times, sustained convergence of eyes in the vertical and/or horizontal direction. These eye movements and associated EOM contraction can be another cause of increased IOP with smartphone usage.

### Psychophysiological stress

Previous studies on the impact of mental stress on IOP have reported that immediately after exposure to the mental stressor, IOP increase occurred both in healthy controls and open-angle glaucoma patients [10,11]. Psychophysiological stress stimulates the sympathetic input, and IOP is highly influenced by the norepinephrine level increase that arises as part of the sympathetic response [12,13]. Smartphone over-usage and prevalence of depression, anxiety and stress, in fact, are known to be strongly correlated [14]. While the smartphone viewing, consistent reception of new information and concentration on works can act as a sympathetic stimulus, and eventually result in IOP elevation.

### Dry eye

Extended periods of using electronic displays were associated with higher prevalence of dry eye [15,16]. While viewing digital screens, the blink rate is reduced [17,18]. A significantly higher percentage of incomplete blinks also has been observed during the use of an electronic display [19]. The resultant dry eye not only causes ocular discomfort but also can contribute to IOP increase. Dry-eye-related stimulation of the free nerve endings populating the cornea results in afferent nerve impulses through the Trigeminal nerve [20]. Perkins demonstrated that stimulation of the Trigeminal nerve of rabbits cause a rise of IOP in the ipsilateral eye [21]. Although the exact relationship between dry eye and IOP has not been elucidated in clinical setting, there is a possibility that irritation of the corneal nerve endings due to dry eye may affect the IOP fluctuation.

### Neck-flexion posture

In most cases, working with a smartphone tends to be performed below the eye level in the neck flexion posture. Straker et al. reported that a smaller screen requires the user to bend his/her neck more than for a larger monitor [22]. It has been found that neck flexion of an average of 33–45° from the vertical was maintained during the smartphone work [23]. And certainly, body and head position influences IOP [24,25]. IOP was significantly higher when measured with subjects in the neck-flexion posture relative to the neck-extension or the neutral posture [24]. Thus, sustained neck flexion position while reading or writing on a smartphone may contribute to IOP increment.

Interestingly, IOP changes that occurred while using the smartphone were more apparent under low-light condition. Ocular anatomic features such as increased pupil diameter and thickened iris in darkness have been well documented [26]. Low ambient illumination causes more visual fatigue and decreases visual performance during screen work [27]. Ocular-structural changes as well as high contrast between a smartphone screen and the surroundings in dim light can contribute to further IOP elevation compared with high-illumination conditions.

The mean change of IOP was about 2 and 3 mmHg under daylight and low-light conditions, respectively. It could not be determined whether these IOP changes during the smartphone work would increase the risk of glaucoma in normal subjects or not. However, previous reports have shown how short-term IOP-fluctuation increase plays an important role in disease progression in glaucoma patients [28,29]. In particular, in normal-tension glaucoma patients whose baseline and follow-up IOPs are relatively low, increased fluctuations of IOP seem to play a stronger role in glaucoma progression [29,30]. We may deduce that such IOP change during smartphone usage can be of clinical significance for those who are vulnerable to IOP fluctuations. Therefore, future research investigating the clinical implications of IOP changes during smartphone work in both normal subjects and glaucoma patients is warranted.

### Study limitations

The present study’s findings should be interpreted with mindfulness of its limitations. First, depending on the type of smartphone, screen size, resolution and brightness differ. Background and text color, font size and style also can affect the degree of IOP fluctuation. Further, this study protocol was conducted within a limited time interval. IOP elevation can be sustained over the course of longer durations of smartphone usage such as playing games or watching movies. Thus, further research with larger patient cohorts and diverse experimental conditions would be expected to obtain fuller information on the relationship between smartphone usage and IOP. Second, although the examiner closely observed the subjects during the entire duration of the experiment to ensure that each remained attentive to the work, it is possible that their concentration decreased with longer smartphone use. Third, elderly people with presbyopia, open-angle glaucoma patients and narrow-angle subjects might show distinct patterns of IOP change during smartphone usage. Additional studies on these patient groups therefore would be crucial before any firm conclusions can be drawn. Fourth and finally, dark-light changes of iris parameters reflect ethnic differences [26]. In the current study, all of the subjects were East Asian (Korean). Further studies investigating the effect on IOP of working with smartphones as well as ocular-structural changes in other ethnic groups are warranted.

In conclusion, working on a smartphone can transiently increase IOP under both daylight and low-light conditions; further, the IOP increment was faster, and the extent of IOP fluctuation was greater, under the low-light condition. Smartphone users who are concerned about IOP fluctuation are advised to (1) take a break if they read or write on smartphone for more than 5 minutes, and (2) avoid using smartphones wherever possible in dark places.

## Supporting information

S1 Table IOP changes of study participants. (XLSX)
